# *In-situ* behavioural and physiological responses of Antarctic microphytobenthos to ocean acidification

**DOI:** 10.1038/s41598-018-36233-2

**Published:** 2019-02-13

**Authors:** James G. Black, Jonathan S. Stark, Glenn J. Johnstone, Andrew McMinn, Philip Boyd, John McKinlay, Simon Wootherspoon, John W. Runcie

**Affiliations:** 1 Institute for Marine and Antarctic Studies, Castray Esplanade Hobart Tasmania, Hobart, Australia; 2 Australia Antarctic Division, Antarctic Conservation and Management program, 203 Channel Hwy, Kingston, Tasmania Australia; 3 School of Life and Environmental Sciences, University of Sydney, 2006 Sydney, Australia; 4 Aquation Pty Ltd, PO Box 3146, Umina Beach NSW, 2257 Sydney, Australia; 5 College of Life Sciences, Ocean University of China, 5 YuShan Rd, Qingdao, China

## Abstract

Ocean acidification (OA) is predicted to alter benthic marine community structure and function, however, there is a paucity of field experiments in benthic soft sediment communities and ecosystems. Benthic diatoms are important components of Antarctic coastal ecosystems, however very little is known of how they will respond to ocean acidification. Ocean acidification conditions were maintained by incremental computer controlled addition of high *f*CO_2_ seawater representing OA conditions predicted for the year 2100. Respiration chambers and PAM fluorescence techniques were used to investigate acute behavioural, photosynthetic and net production responses of benthic microalgae communities to OA in *in-situ* field experiments. We demonstrate how OA can modify behavioural ecology, which changes photo-physiology and net production of benthic microalgae. Ocean acidification treatments significantly altered behavioural ecology, which in turn altered photo-physiology. The ecological trends presented here have the potential to manifest into significant ecological change over longer time periods.

## Introduction

Ocean acidification (OA) is the change in seawater carbonate chemistry, including a reduction in pH, as a result of the absorption of atmospheric CO_2_ into the oceans^1^. Ocean acidification is predicted to cause a 0.4 decrease in ocean pH and a 190% increase in dissolved inorganic carbon (DIC) by 2100 under a scenario of “business as usual” CO_2_ emissions, as modelled by the IPCC AR5 RCP 8.5^2^. This change is expected to substantially alter the structure of most marine communities^3–6^.

The majority of OA research to date has been on single organisms in laboratory-based studies^7^. In general, these studies indicate that reduced pH will be detrimental to some organisms (some calcifying heterotrophs) but advantageous to others (non-calcifying algae), creating potential “winners” and “losers” in marine communities under future scenarios of higher atmospheric CO_2_^3,4,8^. Whether these single organism responses will translate to actual changes in natural communities, once all ecological interactions are included, is largely untested. If they do, it could result in altered ecosystem services, functioning and community structure^9–11^. Therefore, community-scale OA experiments are an important gap in our current knowledge^12^. Even though the effects of OA are predicted to affect higher latitude waters sooner than elsewhere, there has been little *in-situ* OA research on Antarctic benthic marine ecosystems^13^. The few community-scale OA studies that have utilised an *in-situ* field approach have been undertaken in tropical, deep sea or pelagic ecosystems^7,14,15^, with no published polar research.

Microalgae and most autotrophic algae are generally expected to benefit under OA conditions in comparisons to heterotrophic organisms. This is likely the result of the increase in carbon sources (CO_2_ and HCO_3_−), used for photosynthesis, under OA conditions. However, different species of marine algae show different sensitivities to OA^16,17^, with beneficial and detrimental effects of ocean acidification on photosynthesis reported for different algal species^17–21^. Most studies attribute the different reponses to species specific differences in the efficiency of carbon utilisation of CO_2_ and HCO_3_−, via carbon concentrating mechanisms (CCM)^17,22,23^. Different experimental protocols may also be a factor in some circumstances^17^. While these factors will likely contribute to responses to OA, attributing a generalised response of all algae to OA is confounded by the fact that marine algae (including microalgae) inhabit a diverse range of microenvironments.

Microalgae in the microphytobenthos (MPB) live in a microenvironment that is characterised by much steeper environmental gradients compared to their pelagic counterparts^24^. Gradients of light, pH, O_2_ and DIC change substantially in the MPB after the Diffusive Boundary Layer (DBL). The euphotic zone of MPB mats are typically much thinner than pelagic euphotic zones, yet the microbial activity is higher^25^. This results in rapid and dynamic consumption and production of O_2_ and CO_2_, influencing pH and DIC differentially down the sediment profile^24–26^. These unique micro environmental factors could influence MPB responses to OA, yet there is a paucity of information on MPB OA responses^24^. Additionally the ability of microalgae to deal with OA in the short term may be very different to longer term responses. A review of MPB responses to OA by Marques da Silva, *et al*.^24^ stongly emphazies the need for a better understanding of MPB response to OA.

Microalgae play a vital role in ecosystem functioning^16,21^, especially in the MPB in Antarctic benthic ecosystems, which in low-light habitats under sea ice are the dominant autotroph in the absence of macro algae^27,28^. Microalgae form the basis of many marine food webs and perform key ecological roles in fixing carbon, recycling nutrients, stabilising sediments and contribute to modifying global climates via their role in the carbon cycle^28,29^. While the uptake of carbon by microalgae is essential for their photosynthesis, it is also intrinsically linked to ocean carbon cycling and will interact with OA^23,30–32^. Microalgae have a number of ecologically linked processes that may be altered by OA. For example, MPB photosynthetic yield, primary production and net production drive vital ecosystem services in soft sediment communities, which if altered will have flow on effects to the whole community^33^. Changes to these ecosystem services will be positive or negative dependent largely on whether conditions are more or less preferential for microalgae^34^.

Understanding the physiological preferences for microalgae in the context of carbonate chemistry is essential to understand the likely community shifts in response to future OA conditions^6^. Microalgal physiological preferences can be inferred from a range of quantitative measures such as the effective quantum yield of photosystem II light conversion efficiency (ϕ _PSII_) and biomass changes^21,35,36^. However, inferring short term (i.e. within hours) algal responses to OA would benefit from a behavioural indicator (i.e. avoidance/attraction behaviour). Active behaviour may seem unlikely for single celled marine algae, however many marine benthic diatoms have the capacity to migrate up and down in the sediment in response to external stimuli (i.e. sunlight, temperature, nutrients), to seek out their optimum or beneficial physiological conditions^18,20,24,37^. Photo Active Radiation (PAR) is normally the primary driver of this vertical migration behaviour, which is referred to as the photo tactile response. This behaviour provides an additional measure of microalgal response to environmental conditions that is otherwise difficult to measure in most phytoplankton and macroalgae. Photo Tactile Response (PTR) in benthic diatoms has been used to measure responses to changed environmental conditions in other studies^24,37^, however, it has not previously been used to examine responses to OA *in-situ*.

The purpose of this study was to identify eco-physiologically relevant trends in MPB responses to OA *in-situ*. The specific aims were to investigate if OA alters the already recognized PTR relationship to PAR; and to determine what effect changes in PTR may have on photosynthetic yield and net production. To test this we performed  a series of *in-situ* mesocosm experiments in a sea-ice covered benthic habitat in Eastern Antarctica. Novel technology was used to provide insights into MPB community respiration, photosynthetic and behavioural responses to OA. This study provides the first measurements of *in-situ* effects of ocean acidification on MPB in a community context.

## Results

### Experimental treatments

**Figure 1:**
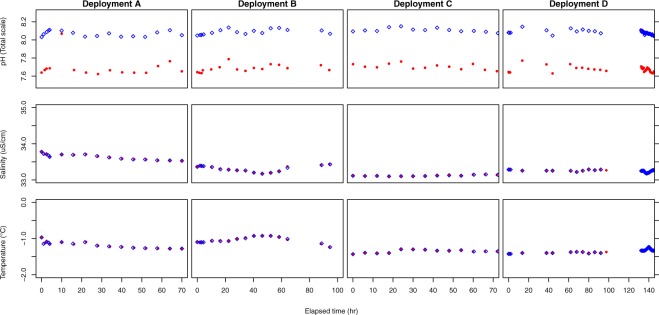
The pH (Total scale), salinity and temperature of control (blue diamonds) and acidified (lower red circles) in deployments (**A**–**D**) (from left to right respectively).

A pH of 0.4 below ambient was maintained in incubation chambers for each deployment (Fig. 1). Other water parameters (i.e. aragonite (Ω) saturation, salinity, temperature) can be found in Table 1 or^38^. Treatment water used in this experiment was pumped from the Antarctic Free Ocean Carbon Enrichment experiment (antFOCE) experimental chambers, acidified treatments maintained a pH offset of 0.3825 ± 0.065, mean Ω_ar_ of 0.62 ± 0.14 and mean *f*CO_2_ values of 912.5 ± 155 µatm over the duration of their experiment. In contrast, water supplied to the control chambers maintained a pH of 8.061 ± 0.044, mean Ω_ar_ of 1.39 ± 0.11 and mean *f*CO_2_ values of 354 ± 42 µatm. Treatment water pH, oxygen and salinity measurements were sampled from the antFOCE sensor system at the time water was pumped from antFOCE treatments into respiration chambers, (Fig. 1). The mean pH of acidified and control treatments, calculated over all deployments, was 7.687 ± 0.01 and 8.082 ± 0.018 respectively. xxx water parameters remained relatively stable between experiments (i.e salinity, temperature) (see Table 1 or^38^).

**Table 1:** Averaged ranges of carbonate chemistry parameters of treatment water used in deployments A-D.

	DIC (µmol kg^−1^)	TA (µmol kg^−1^)	Ω_ar_	*f*CO_2_ (µatm)
**Control treatment**				
Mean range (±s.d.)	2154 ± 35.72	2271 ± 28.48	1.39 ± 0.11	354 ± 42
**Acidified treatment**				
Mean range (±s.d.)	2243.09 ± 54.66	2271 ± 28.15	0.62 ± 0.14	912.5 ± 154.5

DIC = calculated dissolved inorganic carbon; Ω_ar_ = saturation state of aragonite; *f*CO_2_ = fugacity of CO_2_.(data reproduced from^38^).

### Rapid light curves

**Figure 2:**
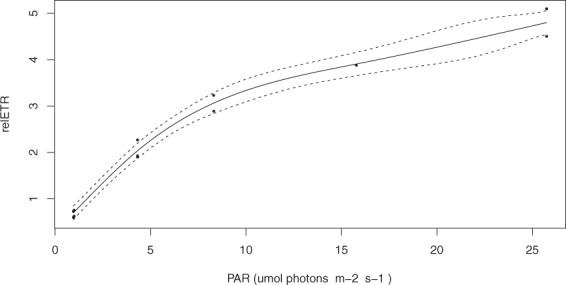
*In-situ* relETR of microphytobenthos under control conditions. A GAM model has been applied to estimate an average response (solid line) with 95% confidence intervals (broken lines).

*In-situ* rapid light curves indicated that the electron transport rate of MPB approached maximium relative electron transport rate (relETR_max_) after 25 μmol photons m^−2^ s^−1^ (Fig. 2).

### Diatom species composition

Microscope counts of diatom cells indicated 15 dominant species (i.e. >1% abundance) (see Table 2). *Trachyneis aspera, Cocconeis fasciolata, Pleurosigma elongatum* and *Cymbella* species were the most common species at the study site with 21, 17,14 and 9% relative abundance respectively. There were a total of 104 species found at this site within the study peroid. There were also low abundances in the MPB community of species commonly refered to as being associated with sea-ice (i.e. *Fragilariopsis cylindrus*).

**Table 2:** Microphytobenthic species at study site.

Microphytobethos species	Relative Abundance (%)
*Trachyneis aspera*	21
*Cocconeis fasciolata*	17
*Pleurosigma elongatum*	14
*Cymbella* sp	9.3
*Achnanthes brevipes*	4
*Amphora libyca*	3.5
*Eucampia antarctica*	3.5
*Pinnularia quadratarea*	3.1
*Synedropsis recta*	2.8
*Pleurosigma obscurum*	2.3
*Navicula directa*	1.8
*Fragilariopsis sublinearis*	1.5
*Actinocyclus curvatulus*	1.4
*Fragilariopsis cylindrus*	1
*Diploneis splendida*	0.7
*Actinocyclus actinochilus*	0.7
*Odentella weissflogii*	0.5
*Pinnularia quadratarea* (variant *constricta*)	0.5
*Cocconeis pinnata*	0.4
*Fragilariopsis kerguelensis*	0.3
*Licmophora belgicea*	0.2
*Thalassionema gelida*	0.1
*Fragilariopsis ritscheri*	0.1
*Manguinea fusiformis*	0.1
*Auricula compacta*	0.1
*Fragilariopsis obliquecostata*	0.1
*Dicyocha* sp	0.1
*Biddulphia areolata*	0.1
*Fragilariopsis rhombica*	0.05
*Melosira adeliae*	0.05
*Pseudogomphonema kamtschatica*	0.05

### Photo tactile response (PTR)

**Figure 3:**
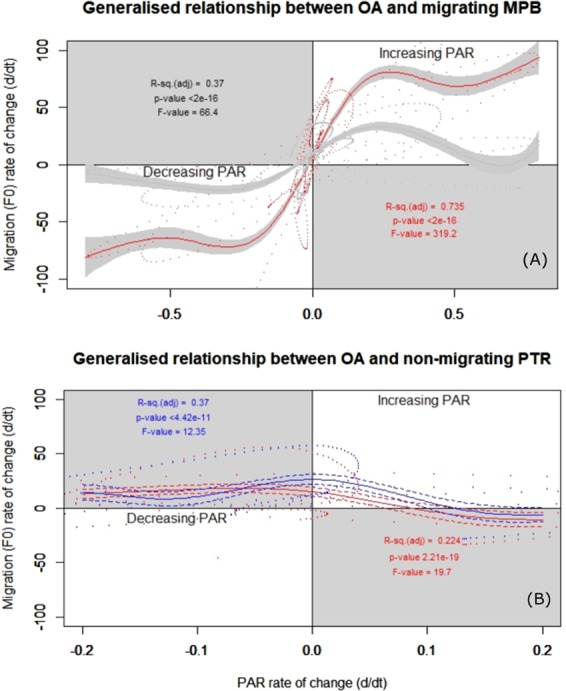
Rate of change of photo tactile response (PTR) in relation to the rate of change of PAR under normal migration behaviour (**A**) and non migrating PTR (**B**). The y-axis is the first derivative (F0^d/dt^) of migration rate, with positive values indicating migration towards the surface, negative values migration away from the surface. The x-axis is the first derivative PAR (PAR^d/dt^), with positive values when PAR increases, negative values for decreasing PAR. The colour of each test statistic (eg. *p*, F-value and Adjusted R-squared) represents it associated treatment. (**A**) Normal PTR in acidified treatment (Red line, with pink solid shaded 95% confidence intervals), normal PTR in control treatment (black line, with solid grey 95% confidence intervals). (**B**) Absent PTR in acidified treatment (red line, with red dashed 95% confidence intervals), absent PTR in control treatments (black line, with dashed black 95% confidence intervals).

Photo tactile response measured simultaneously in both treatment showed a significant relationship to the diel rate of change in PAR (i.e. first derivative of PTR changed with the first derivative of PAR) (*p* = *<2e-16*) (Fig. 3). Stated more simply, as light increased MPB biofilms migrated towards the surface, as light decreased MPB migrated away from the surface. However, acidified PTR displayed a much stronger relationship to PAR^d/dt^ (*p* = *2e-16*, F = 447.8), compared to the relationship in the control treatments (*p* = <*2e-16*, F = 80) (see Fig. 3). The relationship between diel PAR and diel migration decreases in the control treatment  at higher diel PAR rates of change (i.e. higher PAR values). By comparison the acidified diel migration rate maintains a more significant relationship to diel PAR rate even at higher PAR^d/dt^. Deployment C had a substantially different and irregular light regime, which resulted in a noncyclic and non-significant PTR pattern not seen in other deployments. This resulted in the MPB biomass staying at the surface for both treatments, with PTR to PAR being similar between treatments. Additionally as deployment time elapsed beyond 80 hours PTR was also non-significant in both treatments. Therefore, the effect of OA was explored on two MPB behavioural states, where MPB were either displaying normal PTR-PAR migration behaviour or not.

### Diel rate of change of yield ϕ PSII

The effective quantum yield showed a diel rate of change that related to the diel change in PAR (i.e. first derivative of PAR was significantly related to the first derivative of diel yield ϕ _PSII_) (*p* = <*2e-16*). Stated more simply, as PAR increased, the rate of photosynthesis (yield ϕ _PSII_) also increased. The acidified treatment diel yield rate did show a more significant relationship to diel PAR (*p* = <*2e-16*, F-value = 545), compared to the control treatment (*p* = <*2e-16*, F-value = 10).

### Yield ϕ PSII maxima

**Figure 4:**
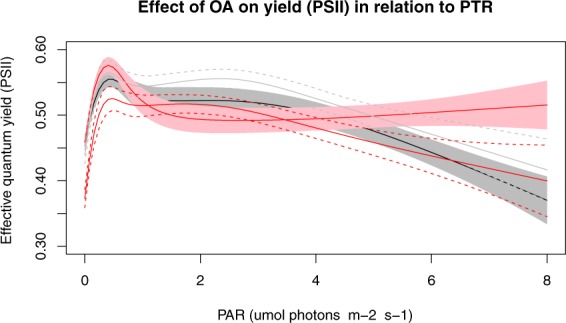
Effective quantum yield (ϕ _PSII_) in relation PAR. Acidified treatment under normal PTR (Red line, with pink 95% confidence interval), control treatment under normal PTR (Black line, with grey 95% confidence interval). Non-PTR in acidified treatment (red line, with red dashed 95% confidence intervals), non-PTR in control treatment (grey line, with grey dashed 95% confidence intervals).

**Figure 5:**
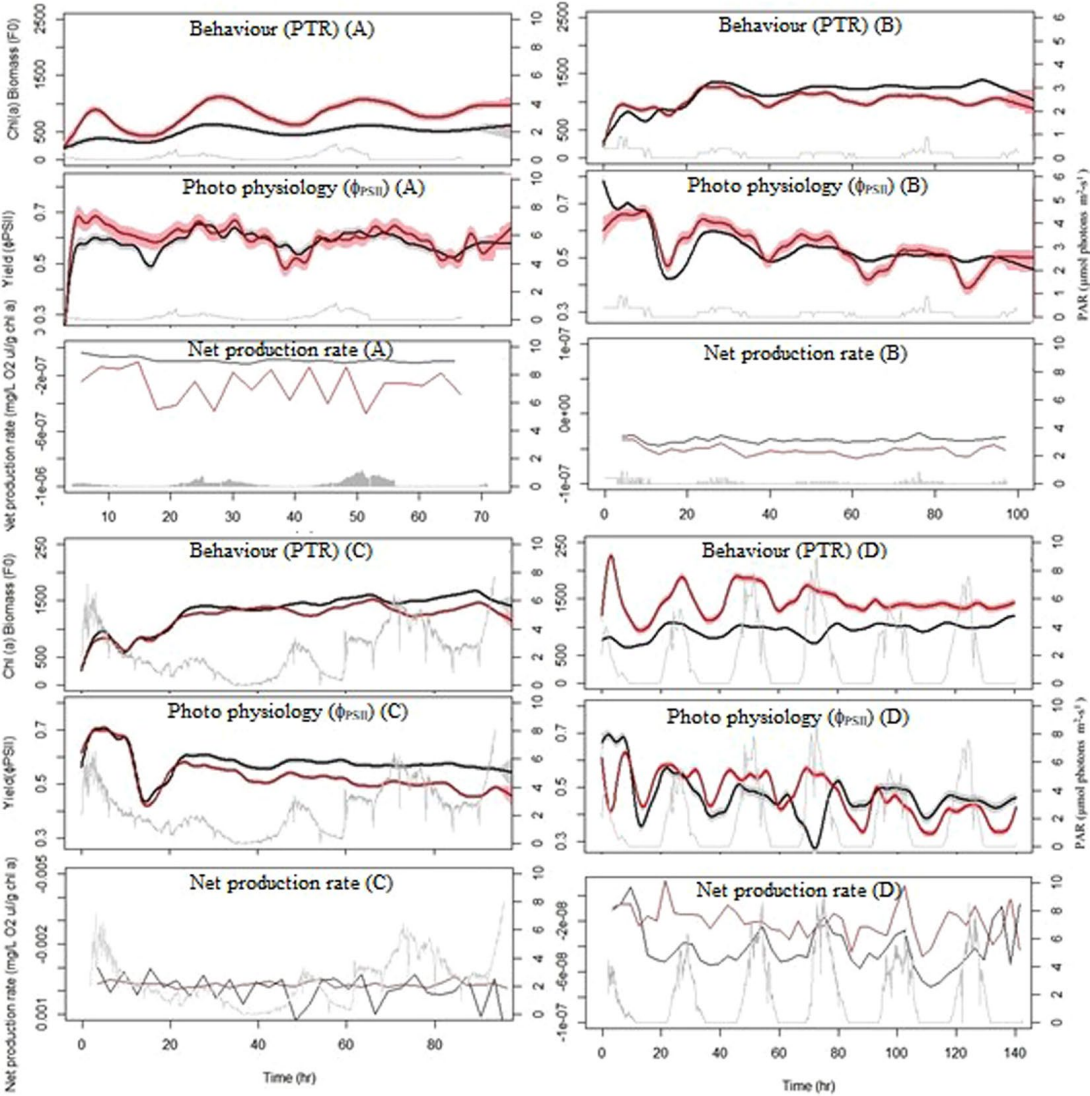
Stacked graphs of PTR, photosynthetic yield (ϕ_PSII_) and net production rate within each experimental deployment (**A**–**D**). X-axis is elapsed time (hours) from start of deployment. Grey lines are Photosynthetic Active Radiation (PAR) with the scale on the right hand side of the graphs in μmol photons m^−2^ s^−1^. Black lines (with grey 95% confidence intervals) are the control treatment and red lines (with pink 95% confidence intervals) are the acidified treatments.

Yield (ϕ _PSII_) increased as PAR increased until ~2 μmol photons m^−2^ s^−1^ in both treatments when MPB were migrating normally. There was no substantial difference between treatments in yield maxima until PAR was higher than ~6 μmol photons m^−2^ s^−1^, at which point control treatment yield then shows lower photosynthetic yield (Fig. 4). This is also observable at PAR maxima in Fig. 5(B and D). However, without PTR the acidified yield (ϕ _PSII_) was trending lower than controls (Fig. 4), demonstrating that without PTR, there is a negative effect of OA on yield (ϕ _PSII_). Under these conditions the PTR-PAR relationship had decreased and yield (ϕ _PSII_) was found to be negatively affected in the acidified treatment compared to the control (Fig. 4).

### Net production rate

Under low PAR conditions (i.e. <10 μmol photons m^−2^ s^−1^) a negative net production of oxygen was observed in all treatments/deployments. There did not appear to be a detectable pattern (from 20 cm above the sediment) of net production matching yield measurement under low PAR in deployments A, B and C (Fig. 4). However, under higher PAR conditions in deployment D (i.e. 8–10 μmol photons m^−2^ s^−1^), a difference in the diel rate of net production was detected in the control, with an increase in the rate of net oxygen production observed in the acidified treatment (Fig. 4D).

### Dark adapted yield (ϕ PSII)

**Figure 6:**
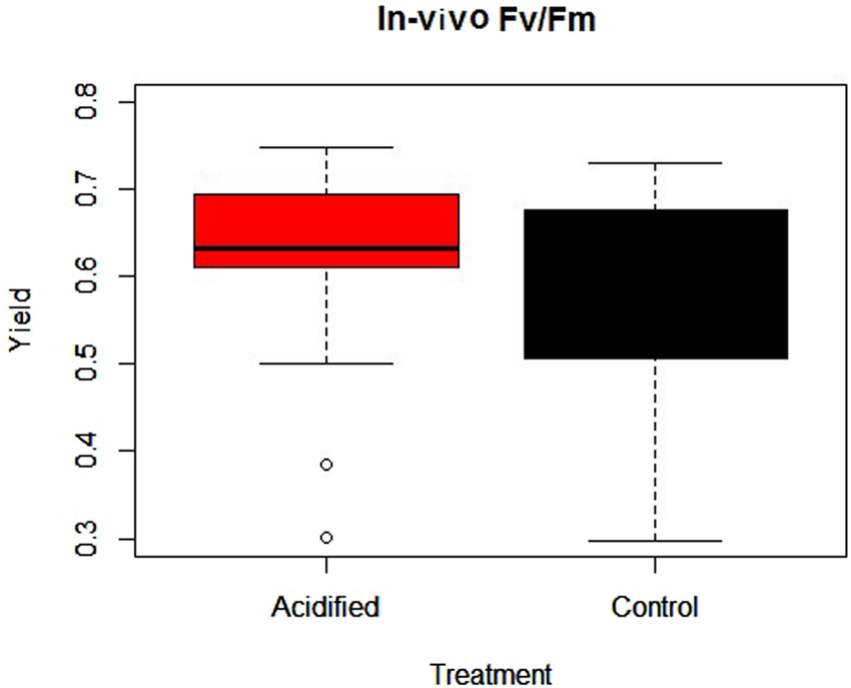
Fv/Fm (ϕ_PSII_) of Control (black box) and Acidified (red box) treatments. Error bars+/− standard error.

There was no significant treatment effect on F_v_/F_m_. (see Table 3, Fig. 6).

**Table 3:** Anova summaries for *in-vivo* yield (F_v_/F_m_) and sediment Chl *a*.

Source	Df	Sum Sq	Mean Sq	F value	P value
***In-vivo*** **yield (F**_**v**_ **/F**_**m**_ **)**					
Treatment	1	0.0236	0.023591	2.461	0.1227
Treatment:Deployment	6	0.1179	0.019644	2.049	0.0751
Residuals	53	0.5081	0.009586		
***Chl a***					
Treat	1	1831068	1831068	10.529	0.00143
Depth	9	14374551	1597172	9.184	3.7e–11
Treat:Depth	9	707018	78558	0.452	0.90459
Residuals	160	27826359	173915

### Vertical sediment profile of Chl *a*

**Figure 7:**
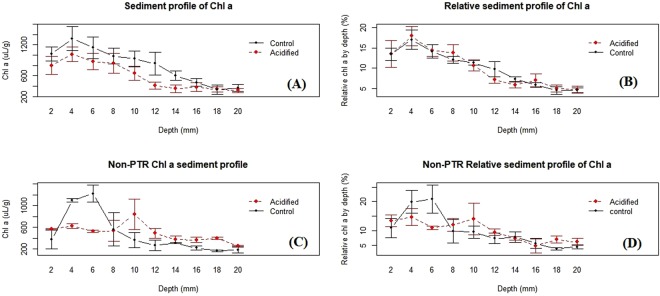
(**A**) Average chlorophyll a sediment profile of PTR deployments (**A**,**B** and **D**). (**B**) Relative Chl *a* concentration at each depth in relation to total core Chl *a* in PTR deployments. (**C**) Non-migrating PTR Chl *a* sediment profiles and (**D**) Non-migrating PTR relative Chl *a*. Control (black lines) and acidified (red lines) treatments in all plots. Error bars+/− standard error of subsamples.

The vertical distribution of Chl *a* in the sediment decreased significantly below 8 mm (Fig. 7), with significant difference between treatments (*p* = *0.001*, n = 4). However, these significant differences need to be considered in the context of starting heterogeneity of MPB biofilms, in addition to these samples being taken at the end of the deployment. The distribution of the Chl *a* at each depth relative the total Chl *a* in each sample core (Relative distribution of Chl *a*) is the more appropriate comparison. While there was no significant difference in relative Chl *a* profiles between treatments, Fig. 7 shows that Chl *a* in acidified treatments have redistributed from deeper to shallower sediment depths (12–14 to 4–8 mm). There was no significant interaction between treatment and sediment depth for Chl *a* (i.e. profiles are similar).

## Discussion

MPB microalgae demonstrated an ability to detect altered DIC states and actively changed their natural photo tactile behaviour to account for OA. Diel yield showed a strong relationship to diel PTR, with PTR and yield (ϕ _PSII_) both having significant relationships with PAR. The changes in PTR under OA corresponded to increases in both diel yield rate and yield (ϕ _PSII_) maxima. This was most pronounced under higher PAR. A number of external abiotic factors (such as irregular PAR cycles) can disrupt the normal PTR patterns. Under these circumstances, when the PTR-PAR relationship was not significant, yield (ϕ _PSII_) was lower in the acidified treatments. This demonstrates the fundamental role of PTR in maintaining photo-physiology and how it interacts with OA. A minimal diel net production relationship (under high PAR only) was also observed.

Photo tactile response displayed a significant PAR driven diel pattern in both treatments (*p* = <*2e-16*) (Fig. 3), which has previously been reported in other ecosystems^18,24,39,40^. However, the acidified treatment had a more significant relationship with PAR (with an F-value of 447.8 and 80 for acidified and control treatments respectively). Photo tactile responses in this study was not correlated with temperature or tide patterns, as found in other systems^40,41^. This is likely due to the very stable environmental conditions in Antarctica compared to other environments (i.e. temperature variation 0.1 °C^−d^, <1 m tidal change and a low flow rate 2–3 m s^−1^). Microalgae were typically found down to 6–8 mm into the sediment, with Chl *a* content decreasing substantially after 10 mm (Fig. 7).

Photosynthetic yield (ϕ _PSII_) showed a significant diel yield relationship to PAR in both treatments (*p* = *<2e-16* in both treatments). This was evidently intrinsically linked to PTR and demonstrated why behaviour of an MPB biofilm is a very important consideration for MPB primary producers under OA.

Yield (ϕ _PSII_) of MPB is proportional to the amount of PAR, nutrients and carbon available for photosynthesis^42^. According to Hancke, *et al*.^43^ the prior PAR regime (i.e. previous ~12 hours) will dictate the current microalgae PTR. Therefore MPB migration through the sediment enables moderation of exposure to PAR, nutrients and carbon in the overlying water (i.e. OA treatment)^24,37^ and will influence MPB primary production.

The majority of studies on microalgae have illustrated either positive or negligible effects of OA on their physiology^16^, with some reporting that many phytoplankton species are insensitive to OA^44,45^. However, our results clearly show that Antarctic MPB are very responsive to OA. The variable response among diatoms is reported to be due to the poor affinity of ribulose 1,5-bisphosphate carboxylase/oxygenase (RuBisCO) for CO_2_ substrates used for photosynthesis, being only ~50% saturated under current CO_2_ levels^16^. Therefore many diatoms operate carbon concentrating mechanisms (CCMs) which concentrate CO_2_ around sites of photosynthesis. In future OA scenarios the DIC available for photosynthesis increases by ~190% (although only 1% of this is CO_2_), and this is expected to saturate RuBisCO with CO_2_ and reduce energy demands for concentrating CO_2_ for photosynthesis. According to Cartaxana, *et al*.^18^, in a crowded MPB community, carbon may be a limiting resource even for organisms with highly efficient CCMs. Carbon limitation may explain why the results presented here indicate an initial increased diel PTR and yield with elevated *f*CO_2_ (i.e. OA conditions), followed by signs of acclimatisation. MPB biofilms can be up to ~1 mm thick, with an area of 1 m^2^ representing a cell density equivalent to ~34,000 litres of high chlorophyll-*a* content open ocean water. Thus it is likely that carbon may be limited in these biofilms, and short term responses in PTR and yield (ϕ _PSII_) are likely to be indicative of the relief of carbon limitation in these MPB biofilms.

The microalgal OA photo tactile response in the current study needs to be considered in the context of microalgae DIC/pH preferences and sediment pH gradients. Microalgae photosynthesis has the capacity to deplete DIC in the few millimetres either side of the DBL, with DIC depletion at the surface and increasing DIC  with depth with strong vertical gradients^24^. We would expect microalgae to seek out higher DIC/low pH regions when photosynthesising, yet avoid the energetic cost of pH homeostasis from low pH/high DIC when in other cellular cycles (i.e. Krebs or xanthophyll cycles).

A greater initial change in PTR and higher photosynthetic yields (ϕ _PSII_) in the OA treatments occurred during periods of higher PTR^d/dt^ (Figs 3 and 5D). In this context it appears that initially, under higher PAR, microalgae are selecting environments with more available carbon and utilising this for increased photosynthetic rates. This corresponds with research by Cook and Røy^46^ who found that increased rates of pore-water advection or addition of HCO_3_ increased photosynthesis. Interestingly, experiments investigating the impact of increasing inorganic CO_2_ on growth have produced a range of positive and negative results, which could be due to physiological variation between species or experimental protocols^16^. There are very few long term studies of OA on photosynthetic yield. However, increased diel yield was only apparent while the PTR-PAR relationship was maintained under acidified treatment conditions in the current study. After 80 hours, we see the acidified PTR decrease and the yield (ϕ _PSII_) similar or lower than the control yield (ϕ _PSII_) (Figs 4 and 5D). We suspect that MPB would be able to utilise extra DIC associated with OA for photosynthesis up until they have reached their maximum diel yield capacity. Any extra DIC not consumed by photosynthesis would then contribute to pH decreases (i.e. ocean acidification). Thus it is possible that negative diel yield after 80 hours is in part due to: (1) CO_2_ saturated MPB biofilms not utilising extra DIC which then contributes to a lower pH in the diffusive boundary layer, which then requires them to increase proton pumps to maintain the intracellular pH homeostasis; or (2) an absence of PTR observed after 80 hours in the current study resulting in non-photochemical quenching (NPQ). If MPB are photo inhibited (NPQ) due to lack of PTR, then consequently they would not be using the extra DIC for photosynthesis and we would expect condition (1) above to influence yield. For example, deployment (C) had variable and non-cyclical PAR cycle events resulting in MPB staying at the sediment surface. This demonstrated that, when the diatoms remained on the surface with irregular PAR and maximised their exposure to OA and PAR, it resulted in negative photosynthetic yield (ϕ _PSII_). It is possible that this is due to high non-photochemical quenching (NPQ) of the MPB as they receive more PAR than could be processed. Also, as stated above, if MPB are not actively using DIC for photosynthesis then it will contribute to decreasing pH in surrounding water bodies (i.e. in the Diffusive Boundary Layer). These findings are consistent with the findings of Hoppe, *et al*.^47^ who illustrated that irregular PAR intensities strongly modulated the effects of OA on marine phytoplankton. They demonstrated that irregular/dynamic PAR reduced growth and strongly altered the effects of OA on primary production, being unaffected by elevated *f*CO_2_ under constant PAR. Positive effects of OA on yield (ϕ _PSII_) observed in the current study are assumed to be due to the ability to self-regulate exposure to OA and PAR through PTR. However, if this ability is impaired (i.e. no sediment migration) then we assume that MPB have to deal with lower pH without photosynthesis to buffer lower pH/high DIC (due to NPQ).

Previous studies on algae under OA scenarios have generally not found any differences in photosynthetic yield^48–50^. However, the majority of studies investigating OA effects on yield (ϕ _PSII_) have used Fv/Fm^49–52^ rather than light-adapted samples or diel effective quantum yield (ϕ _PSII_) measurements. There is a fundamental physiological difference in what yields signify under dark or light-adapted methods. Dark adapted yield (Fv/Fm) represents the maximum capacity of the photosystem to convert PAR energy into charged states and direct this into the photochemical pathway. This can be used to test the ability of photosystem (II) to function (i.e. if (ϕ _PSII_) is damaged by a stimulus/toxin), with negative dark adapted yield (Fv/Fm) responses typically seen under toxin and nutrient stress^53^. In comparison, diel light-adapted yield is testing photosystem (II) rate of functioning at natural irradiances and is ideal for comparative OA field studies^53^. Therefore we would emphasize that in the context of OA and ecosystem responses, it is more appropriate to be examining diel light-adapted yield (ϕ _PSII_), especially in the field. In the current study we examined both light and dark-adapted yield (ϕ _PSII_). In contrast to what we observed with light-adapted-yield (ϕ _PSII_), when we examined dark adapted Fv/Fm (20 minutes) we observed no significant difference between treatments (Fig. 6).

Photosynthetic organisms have a high capacity to modify pH, with evidence that pH is regulated at the cell/water interface^25,54^. Therefore we would not expect to see damage to photosystem (II) from more acidic conditions, provided they have the ability to buffer this at the cell/water interface using extra DIC for photosynthesis. So it is not surprising that there was no difference in dark adapted yield (ϕ _PSII_) in this study or previous studies.

Photosynthetic yield (ϕ _PSII_) correlates with oxygen production in most algae^27,55^. In the current study, natural communities of heterotrophs and autotrophs were enclosed in the same respiration chambers in an ecological setting, therefore we can only report net community production rather than individual contributions of MPB to net production in relation to increased yield (ϕ _PSII_). Furthermore, the DO sensors were 20 cm above the sediment in a 27 litre volume of water. It is expected that this reduced our detection limit substantially. Repeated 6 hour respiration incubations were always negative in net production. This indicates two potential situations: (1) a higher biomass of heterotrophs relative to microalgae; or, (2) the irradiance was not sufficient to reach the E_c_ (E^k^), where oxygen production exceeds consumption. The present study observed a corresponding diel pattern in the net production rate and yield under higher PAR conditions in controls (Fig. 5D). This indicates that at higher PAR levels our DO detection limit was adequate. In this same deployment the acidified treatment had a higher net production rate. Therefore we are confident that the higher net production rates observed in acidified treatments in deployment D are related to corresponding higher yield values.

Heterotrophic organism metabolic functioning (i.e. respiration) will reduce net production and needs to be considered in the context of our results. Metabolic up-regulation or depression in response to OA has been documented in many marine species, however some species show no change in metabolism^56,57^. Therefore, increases in net production under OA might be partially attributed to MPB increasing O_2_ production, or a change in heterotrophic O_2_ consumption.

Coastal pH often displays a strong short term diel rather than a static pH pattern. The short term responses in the current study are a valuable insight into MPB’s capacity to deal with short term changes in carbonate chemistry conditions. The results from this study demonstrate that MPB communities are not insensitive to higher DIC and lower pH associated with OA. Any changes in net production and photosynthetic yield (ϕ _PSII_) will have flow on effects via changes to dissolved oxygen levels and primary production as increased biomass at the base of the food web^4,58^. With the global importance of MPB in supplying ecosystem services and their role in carbon cycling, any change in their physiology or ecology due to environmental changes such as ocean acidification could have potentially widespread consequences. Longer term ocean acidification induced changes in MPB physiology will be dependent on the following factors; (1) ability to migrate effectively through the sediment; and (2) ability to utilise extra DIC associated with OA for photosynthesis.

## Materials and Methods

### Study site

Field experiments were conducted at O’Brien Bay, near Casey Station, East Antarctica (66.311500° S, 110.514216° E) between 1^st^ December 2014 and 1^st^ March 2015. The site was characterised by 2.5 m thick multiyear sea ice and the depth ranged from 12 to 14 m. The dominant primary producers at the study site were microphytobenthos (MPB) and sea ice algae, with a complete absence of macroalgae due to the low PAR conditions at the site, which rarely exceeded 10 μmol photons m^−2^ s^−1^ at the seafloor (see^38^). The MPB at the site formed a dense mat (~0.5–1 mm) on top of the sediment. The benthic marine communities at the site include a range of mobile macrofauna including burrowing anemones, asteroids, holothurians, and filter feeding invertebrates (sponges, ascidians, polychaetes). A previous study indicated significant spatial variation in infaunal communities at scales as small as 10 m, but such differences were small compared to those evident at larger spatial scales^59^. The treatments in the current study were side by side (i.e. <75 cm apart), therefore we make the assumption that heterotrophic and autotrophic communities were relatively similar between treatments, within deployments. A general description of the diatom communities present at the site can be found in Polmear, *et al*.^60^ or supporting species list (Table 2). Snow cover and thickness on the sea ice varied over the experimental period, resulting in different PAR exposures for each experiment. Water flow rate at the site was 2–3 cm s^−1^. Temperature, alkalinity, pH, salinity, dissolved oxygen, were recorded continuously and were considered stable during the study period (see^38^).

### Experimental design

Experiments were conducted in two sealed respiration incubation chambers with a volume of 27 litres (Submersible Photosynthesis-Respiration System, Aquation Pty Ltd, Umina Beach, Australia), deployed on the seabed over patches of MPB. The chambers were made of UV-transparent acrylic with 25 cm diameter (chamber diameter) stainless steel sleeves on the base for insertion into the sediment. The chambers pumped acidified or control water from a larger long-term CO_2_ enrichment experiment (Antarctic Free Ocean Carbon Enrichment antFOCE) via automated pumps (see^38^). A five hour acclimation period was used to introduce water from the antFOCE system gradually, with an increasing ratio of treatment: ambient water pumped into chambers every 5 minutes.

Chamber deployment sites were within 15 m of the antFOCE experimental site. Treatments were placed side by side to reduce spatial differences in species abundance and irradiance. All experiments were conducted at a depth of 12–14 m. Acidified treatments maintained a pH reduction of 0.4 from ambient, in line with IPCC predictions under business as usual emissions scenarios for the year 2100. This reduction in pH, which equates to atmospheric CO_2_ levels of approximately 936 ppm^2^, is hereafter referred to as the acidified treatment. Control treatments maintained an ambient pH level of 8.019 to 8.130, hereafter referred to as the control treatment. These experiments were repeated 4 times (deployments A-D), for periods between 72–144 hours, in the same area between January and February 2015. Figure 1 shows the pH offset for each deployment, as well as other water quality parameters (i.e. salinity and temperature), which did not change between deployments. The Submersible Photosynthesis-Respiration System comprised two chambers, which each included a Shutter Fluorometer fluorescence sensor (Aquation Pty Ltd, Umina Beach, Australia) to record fluorescence parameters and a dissolved oxygen (DO) probe (*In-Situ* Inc. Fort Collins, Colorado, USA) and two LI-192SA planar PAR sensors (Li-COR Inc., Lincoln, Nebraska, USA) to record environmental variables. These sensors were coupled to an underwater programmable data logger (Submersible Datalogger, Aquation Pty Ltd, Umina Beach, Australia) which recorded measurements over the following time scales: ϕ _PSII_ (F0 and Fm) every 30 minutes, Dissolved oxygen (DO) every 5 minutes, PAR every 5 minutes, temperature every 5 minutes. The chambers were flushed (for six minutes) every 6 hours with appropriate treatment or control water.

### Chlorophyll fluorescence measurements

All *in-situ* Pulse Amplitude Modulated (PAM) fluorescence measurements (ϕ _PSII_) are based on samples that had been exposed to natural irradiances, followed by 2–4 seconds of shading as the sensor closes into position prior to measurement. This brief interval of low to zero PAR had no detectable influence on photo acclimation, hence F_0_ and F’q are reported as steady state minimum (F_0_) and maximum (F_m_) fluorescence (see^61^ for PAM fluorescence terminology). Yield ϕ _PSII_ was determined by the equation: yield ϕ _PSII_ = (F_m_′ - F′)/F_m_′. Maximum photochemical quantum yield of PSII dark-adapted yield (F_v_/F_m_) was determined after 20 minutes of dark adaption and was calculated as ϕ _PSII_ = (F_m_ − F_0_)/F_m_.

The minimum fluorescence value (F_0_) was used as a proxy for biomass of MPB biofilms at the surface. This technique was validated by Serôdio *et al*. (2006) and was used to determine MPB sediment migration patterns. Concurrent time lapse photography (Cannon EOS600D and using a digisnap 2000) visually confirmed that the diel migration of MPB corresponded to a measurable F_0_ diel pattern (unpublished data). Changes in F_0_ under different PAR environments are expected to be minimal and consistent across both treatments. At the start of each deployment the Aquation software (Aquation Direct V2.0, Umina Beach, Australia) automatically adjusted the gain and auto zero. Rapid light curves (RLC) were used to calculate the relative Electron Transport Rate (relETR), which was calculated as: relETR = (Φ_PSII_ × PAR) (where values of absorbance and PSI:PSII are assumed to be in unity). Rapid light curves (RLCs) consist of eight steps of increasing actinic PAR for 10 seconds, with a saturating pulse measurement at the end of each actinic step. In all figures and tables the time of day is presented as Coordinated Universal Time (UTC + 8 hours) or elapsed time since beginning of deployment.

### Net production

Dissolved oxygen sensors recorded every 5 minutes in each chamber. Linear regressions were applied to discrete blocks of data (180 minutes) with the slope coefficient used to measure the rate of change of Net production. These values were standardised by total microalgal sediment Chl *a* content.

### Sample Collection

At the end of each experiment, five core samples for species identification and biomass and one core sample for grain size analyses were taken using PAR-proof syringes (3 cm × 10 cm) and carefully extracted from the sediment, placed in a dark container and returned to the surface. Dark acclimated yield measurements (Fv/Fm) were taken for surface MPB after 20 minutes and then the cores were sliced every 2 mm down to 20 mm in the field. Each 2 mm section was immediately placed in the dark in cryotubes at −20 and divided in half for species identification and chlorophyll *a* (Chl *a*). These sample were then transported back to a −80 °C freezer (within 3 hours). Samples used for species identification were preserved in glutaraldehyde (4%) and stored at ~10 °C for later analyses. The remaining section of each core was placed on ice and transported back to a -80 °C freezer. All analyses were conducted at the Australian Antarctic Division (Hobart, Australia) or the Institute for Marine and Antarctic Studies laboratories (University of Tasmania, Hobart).

### Environmental water and carbonate chemistry analysis

Sea water pH, salinity and temperature were recorded every 30 minutes via the antFOCE system^38^. Dissolved inorganic carbon and total alkalinity samples were taken regularly to quantify the carbonate chemistry of treatments. The CO2SYS function in MATLAB (version 9.4.0) was used to calculate pH (from DIC, TA data). The pH (total scale) and salinity in each antFOCE chamber was determined at the time when water was pumped into the deployment chambers (Fig. 1).

### Chlorophyll *a* analysis

Sediment pigment samples were extracted in methanol (99% HPLC grade sigma alrich) for 18 hours then analysed by fluorometer (Turner 10 AU, San Jose, California, USA). The fluorometer was calibrated with a Chl *a* standard (Sigma Alrich Chl *a*) and a solid standard (Turner) was used between each measurement to determine drift in signal. Hydrochloric acid (0.1%) was added to determine the phaeophytin component of each sample. These values were standardised by sediment weight.

### Species identification

Samples for MPB species identification were centrifuged at low revolutions (300 rpm) for 15 minutes (1 gram in 10 ml of filtered seawater) to remove the larger sediment particles. The MPB cells were then diluted in 50 ml of filtered seawater. Subsequent aliquots (1 ml) of this dilution was then analysed on a Flowcam (Benchtop B3 series) with associated software (Visual spreadsheet, version 3.4). Only cells that had complete unbroken frustules with intact cell contents were counted. Species were identified according to taxonomic species keys in Scott and Marchant^62^. Total abundance was calculated from the cell counts of each species as a percentage of total cells counted.

### Data analysis

#### Extracting first derivatives from individual deployment models

Separate generalized additive model (GAMs) were fitted to each deployment response (i.e. diel F_0_, ϕ _PSII_ and PAR) using elapsed time since the start of the deployment as the independent variable. Models assumed a Gaussian family for the response, with an identity link function, which proved adequate for these data. Each response variable was regressed against elapsed time of deployment using an adaptive P-spline with 20 basis functions, and 5 penalty basis functions determining the degree to which that flexibility was allocated across the covariate space^63^. This type of adaptive smooth allows the degree of smoothing to vary along the covariate range, as dictated by the variability in the response. An optimal degree of smoothing was determined via cross-validation with final model fit and residuals assessed as suggested by Wood^63^ (via k-index, adjusted R^2^, and standard residual diagnostics). A temporal influence identified from these GAM model residuals directed us to allow effective degrees of freedom to be set at individual time points (using bs = “ad”). The final models had R-squared values between  0.87 +/− 0.09 (1 sd), unless otherwise stated in figures. The first derivative (d/dt, i.e. the slope) for each response variable (diel F_0_, ϕ _PSII_ and PAR) in each deployment model above was extracted for 200 equally spaced segments on the predictor axis (elapsed time). This was carried out using the package Deriv R^64^ in R. The first derivative, denoted here as^d/dt^ represents the rate of change of each variable over the same elapsed time segment for each deployment. The function ‘SignifD’ in R was used to identify significant rates of change in PTR^d/dt^ over time, with no PTR within a 12 hour period classed as non-migratory MPB. The data was then grouped into two separate categories (migrating MPB and non-migrating MPB). The first derivative data from the migrating deployments (A, B and D) were merged into a single dataset for analysis. The non-migrating MPB (deployment C) was examined separately.

#### Examining trends of diel F_0_ and *ϕ*_PSII_ rates of change

To examine how the rate of change of PAR and the rate of change of MPB migration co-varied, we regressed the first derivatives of these variables in a combined dataset model. These GAMs were then used to identify the relationship between the rate of change in PAR and the rate of change in photo tactile behaviour or yield (ϕ_PSII_) between treatments. Models assumed a Gaussian family for the response, with an identity link function, which proved adequate for these data. Each response variable was regressed against the corresponding first derivative of PAR using an adaptive P-spline with 8 basis functions, and 5 penalty basis functions^63^. Both treatments were allocated the same basis function and model parameters. The model fit, residuals and effective degree of freedom were checked as above and as suggested by Wood^63^.

To determine the rate of change of net production, coefficients of the regression of oxygen consumption in discrete 180 minute data blocks were determined. R squared values for fitted regressions of these coefficients were consistently high (>0.9). We make two assumptions with calculation of net production rates: the first is that heterotrophic community members do not change their metabolism under OA; and the second is that the heterotrophic communities are similar between treatments.

Fv/Fm was analysed using a nested ANOVA with deployment nested within treatment (n = 4).

Vertical sediment Chl *a* profiles were analysed on raw Chl *a*, and the percentage of Chl *a* present at each depth relative to the total Chl *a* in each core (relative Chl *a*). The data was grouped in migrating and non-migrating MPB biofilms. Core Chl *a* data was analysed by a two-way ANOVA with depth, treatment (n = 4).

All statistical analyses were carried out in R (version 3.2).

## Data Availability

10.4225/15/5a28b5eb04f71 and 10.26179/5c1827d5d6711
